# Connecting Generations in Senior Housing: Promising Practices and Challenges

**DOI:** 10.1093/geroni/igaa057.2604

**Published:** 2020-12-16

**Authors:** Nancy Henkin

**Affiliations:** Generations United, Merion, Pennsylvania, United States

## Abstract

Senior housing can be an ideal platform for high quality intergenerational programming. Often older adults who move to congregate housing settings experience feelings of loneliness and a loss of purpose. Creating long term partnerships with educational and youth-serving organizations can help senior housing providers expand residents’ social networks and create meaningful civic engagement opportunities. A 3-year national initiative involving an environmental scan of intergenerational practice in senior housing communities, the development of a toolkit for senior housing providers, and the piloting of intergenerational partnerships and programs in six national housing communities was conducted by Generations United and Leading Age and supported by the Retirement Research Foundation. Promising practices, challenges, and lessons learned from this initiative will be shared and strategies for “scaling” this work will be discussed.

